# New and Interesting Pine-Associated Hyphomycetes from China

**DOI:** 10.3390/jof10080546

**Published:** 2024-08-03

**Authors:** Wen-Hui Tian, Yan Jin, Yue-Chi Liao, Turki Kh. Faraj, Xin-Yong Guo, Sajeewa S. N. Maharachchikumbura

**Affiliations:** 1 Center for Informational Biology, College of Life Science and Technology, University of Electronic Science and Technology of China, Chengdu 611731, China; wenhuitian@std.uestc.edu.cn (W.-H.T.); jinyan4759@163.com (Y.J.); yuechicoco@163.com (Y.-C.L.); 2 Department of Soil Science, College of Food and Agriculture Sciences, King Saud University, P.O. Box 145111, Riyadh 11362, Saudi Arabia; talasiri@ksu.edu.sa; 3 College of Life Science, Shihezi University, Shihezi 832000, China

**Keywords:** Dothideomycetes, phylogeny, rare taxa, Sordariomycetes, taxonomy

## Abstract

Pine trees play a crucial role in the forests of Sichuan Province, boasting rich species diversity and a lengthy evolutionary history. However, research and investigation on fungi associated with pine trees are insufficient. This study investigated the diversity of hyphomycetes fungi associated with pine trees in Sichuan Province, China. During the survey, we collected five specimens of hyphomycetes from branches and bark of species of *Pinus*. Five barcodes were selected for study and sequenced, including ITS, SSU, LSU, *TEF1*, and *RPB2*. Morphological examination and multi-locus phylogenetic analyses revealed three new species, viz. *Catenulostroma pini* sp. nov. within Teratosphaeriaceae, *Kirschsteiniothelia longisporum* sp. nov. within Kirschsteiniotheliaceae, *Sporidesmiella sichuanensis* sp. nov. within Junewangiaceae, and two known species, *Paradictyoarthrinium diffractum* and *P. hydei* within Paradictyoarthriniaceae, which are the new host records from *Pinus* species. *Catenulostroma pini*, distinguished from other species in the genus by its unique morphology, has three conidial morphologies: small terminal helicoconidia, scolecoconidia with many septa, and phragmoconidia conidia. *Kirschsteiniothelia longisporum* has longer spores when compared to the other species in the genus. According to phylogenetic analysis, *Sporidesmiella sichuanensis* formed an independent clade sister to *S. aquatica* and *S. juncicola*, distinguished by differences in conidial size.

## 1. Introduction

Hyphomycetes are a polyphyletic group of fungi that lack fruiting bodies (conidiomata), have hyphae that may be immersed in the substrate or not, and where sporulation mainly occurs on differentiated septate hyphae [1,2]. Hyphomycetes represent the asexual forms of many fungal species and are highly diverse, with more than 2265 genera and 13,800 species reported worldwide [2,3]. Hyphomycetes are widely distributed and can be saprophytic in freshwater, marine, and terrestrial ecosystems, or they can parasitize animals and plants as pathogens [4,5,6,7]. Many of the fungi in this group are aquatic and characterized by the production of conidia that are passively discharged from the hyphae, facilitating dispersal [8,9,10]. Since most conidia are characteristically shaped, species can often be identified by morphology, a common practice in the study of dematiaceous hyphomycetes [11,12,13,14]. However, since species may have undergone convergent evolution in morphology, phylogenetic analysis should also be incorporated into classification.

*Catenulostroma* (Teratosphaeriaceae, Dothideomycetes) was introduced by Crous et al., with the type *C*. *protearum*, which was previously placed in the genus *Trimmatostroma* [15]. *Trimmatostroma* and *Catenulostroma* are morphologically similar; however, phylogenetically, they appear as distinct genera, with the type species of *Trimmatostroma* belonging to the order Helotiales [15]. The characteristics of *Catenulostroma* are hypha-like conidiophores and conidia in basipetal chains [15,16]. Species of this genus include saprobic and pathogenic fungi that are occasionally isolated from opportunistic human diseases [15].

*Kirschsteiniothelia* (Kirschsteiniotheliaceae, Dothideomycetes) was established by Hawksworth to accommodate the type species *K. aethiops* [17]. *Kirschsteiniothelia is* a holomorphic genus with two types of asexual morphs: dendryphiopsis-like and sporidesmium-like [18]. Combining morphological and molecular evidence, *Dendryphiopsis* was confirmed to be an asexual of *Kirschsteiniothelia* [19,20]. Subsequently, Wijayawardene et al. recommended using *Kirschsteiniothelia* over *Dendryphiopsis* [21]. Members of this genus are primarily saprophytes found on dead or decaying wood in freshwater and terrestrial habitats and have occasionally been associated with mycoses [8,18,20,22,23,24,25,26,27]. Currently, there are 49 epithets are listed in Index Fungorum (http://www.indexfungorum.org; 7 July 2024).

*Paradictyoarthrinium* (Paradictyoarthriniaceae, Dothideomycetes) was introduced as a monotypic genus with *P. diffractum* as the type species [28]. The genus is characterized by gregarious, black, powdery colonies and macronematous conidiophores with asymmetrically and unevenly dictyoseptate, muriform, subglobose to ellipsoidal conidia [29,30]. Currently, there are only five species in the *Paradictyoarthrinium*, viz. *P. aquatica*, *P. diffractum*, *P. hydei*, *P. salsipaludicola* and *P. tectonicola*, of which *P. aquatica, P. diffractum* and *P. hydei* have records from China [28,29,30,31]. *Paradictyoarthrinium* species are primarily saprophytes found on decaying wood in terrestrial, freshwater, and marine environments [28,29,30,31].

Kirk erected *Sporidesmiella* (Junewangiaceae, Sordariomycetes) with the type *S. claviformis* and introduced two newly described species and four new combinations [32]. *Sporidesmiella* is mainly characterized by clavate or obovoid to cuneate, rounded or coronate at the apex, distoseptate conidia, seceding schizolytically from monoblastic, integrated, terminal, annellidic or rarely sympodially extending conidiogenous cells [32,33,34]. Species of *Sporidesmiella* are saprophytes found on leaves, submerged wood, or decaying wood in freshwater and terrestrial habitats [10,33,34,35,36,37]. To date, there are 54 epithets of *Sporidesmiella* (http://www.indexfungorum.org; 7 July 2024).

Pine trees, the primary community-forming species of coniferous forests in the Northern Hemisphere, constitute the largest group of extant gymnosperms and have a long evolutionary history [38]. Pine trees are a crucial component of the forests in Sichuan Province. This Province has a diverse climate, complex terrain, and abundant pine species, providing a wide range of habitats conducive to the growth and diversification of fungi, resulting in vast fungal diversity. Despite numerous new taxa being reported in recent years, our understanding of them remains limited [39,40,41]. We regularly conduct fungal diversity surveys in Sichuan Province and investigate the taxonomy of fungi associated with *Pinus* spp. During the study, five hyphomycetous fungi were collected from *Pinus* spp. Through multi-locus phylogenetic analysis and morphological examination, we identified these five collections as three new species: *Catenulostroma pini* sp. nov., *Kirschsteiniothelia longisporum* sp. nov., *Sporidesmiella sichuanensis* sp. nov., and two new host records of *Paradictyoarthrinium* from *Pinus* spp.

## 2. Materials and Methods

### 2.1. Sample Collection, Morphological Examination and Isolation

We surveyed the fungal diversity of Hyphomycetes on *Pinus* spp. in Sichuan Province, China, from March to August 2023. The specimens were taken into the laboratory in paper envelopes for examination. Microscopic characters were observed and recorded using a Nikon SMZ800N stereo microscope equipped with a Nikon DS-Fi3 camera (Nikon Corporation, Tokyo, Japan) and a Nikon ECLIPSE Ni-U microscope (Nikon Corporation, Tokyo, Japan) fitted with a Nikon DS-Ri2 microscope camera (Nikon Corporation, Tokyo, Japan). Measurements were conducted using the Nikon NIS-Elements Documentation Imaging Version 5.21.00 (Nikon Corporation, Tokyo, Japan). All photographs were processed using Adobe Photoshop version 22.0 (Adobe Inc., San Jose, CA, USA). Single conidium isolation was made following the method described by Senanayake et al. [42]. Germinated conidia were individually transferred to potato dextrose agar (PDA) media plates and incubated in the dark at 25 °C. Culture characteristics were examined and recorded regularly after 1–3 weeks.

The holotype specimens were deposited in the Herbarium of Cryptogams Kunming Institute of Botany Academia Sinica (HKAS), Kunming, China, and all specimens were deposited in the Herbarium of the University of Electronic Science and Technology (HUEST), Chengdu, China. The living ex-type cultures were deposited in the China General Microbiological Culture Collection Center (CGMCC) in Beijing, China, and all living cultures were deposited in the University of Electronic Science and Technology Culture Collection (UESTCC), Chengdu, China. The taxonomic descriptions of the new taxa have been deposited in MycoBank.

### 2.2. DNA Extraction, PCR Amplification and Sequencing

Fungal genomic DNA was extracted from mycelia using the Trelief^TM^ Plant Genomic DNA Kit (TSINGKE Biotech, Shanghai, China) according to the manufacturer’s protocol. For *Sporidesmiella sichuanensis* specimens (HKAS 136267), obtaining a culture was not feasible, necessitating the direct extraction of DNA from fruiting structures using the method used by Wanasinghe et al. [43]. Five loci, the nuclear ribosomal internal transcribed spacer (ITS: ITS1-5.8S-ITS2), the nuclear ribosomal small subunit rRNA (SSU), the nuclear ribosomal large subunit rRNA (LSU), the partial translation elongation factor 1-alpha (*TEF1*), the partial second largest subunit of RNA polymerase II (*RPB2*), were selected for study and amplified by polymerase chain reaction (PCR). The corresponding primer pairs and PCR conditions are listed in Table 1. The final reaction volume of the PCR reagent was 25 µL, containing 2 µL of DNA template, 1 µL each of the forward and reverse primer, 8.5 µL of double-distilled water (ddH_2_O), and 12.5 µL of 2×Flash PCR MasterMix (mixture of DNA Polymerase, dNTPs, Mg^2+^ and optimized buffer; CoWin Biosciences, Taizhou, China). The PCR products were visualized by 1% agarose gel electrophoresis. Sanger sequencing was conducted by Tsingke Biological Technology (Beijing, China). Newly generated sequences were deposited in GenBank, and the accession numbers are listed in Table 2, Table 3, Table 4 and Table 5.

### 2.3. Phylogenetic Analyses

According to the corresponding Sanger sequencing chromatograms, misleading data from the ends of raw sequencing fragments were manually trimmed and assembled into consensus sequences using SeqMan Pro version 7.1.0 (DNASTAR, Inc., Madison, WI, USA). Barcode sequences of all species (Table 2, Table 3, Table 4 and Table 5) were downloaded from the NCBI nucleotide database using the R package Analysis of Phylogenetics and Evolution 5.0 (APE, http://ape-package.ird.fr, 7 July 2024) [52].

The multiple sequence alignments were conducted using MAFFT version 7.310 [53] with options “--adjustdirection --auto”, and the alignment files were further trimmed using trimAl version 1.4 [54] with the option “-gapthreshold 0.5”, which only allows 50% of taxa with a gap in each site. The best-fit nucleotide substitution models for each locus were selected using ModelFinder version 2.1.1 [55] under the Corrected Akaike Information Criterion (AICC). All sequence alignments were combined using an in-house Python script.

Maximum Likelihood (ML) and Bayesian analysis (BI) were conducted based on individual and combined datasets. Four phylogenetic trees were constructed by multi-locus phylogenetic analyses. The first tree represents the phylogenetic analysis of *Catenulostroma*, the second tree represents the phylogenetic analysis of *Kirschsteiniothelia*, the third tree represents the phylogenetic analysis of *Paradictyoarthrinium*, and the fourth tree represents the phylogenetic analysis of *Sporidesmiella* within the Junewangiaceae. ML phylogenetic trees were obtained using the IQ-TREE version 2.0.3 [56], and the topology was evaluated using 1000 ultrafast bootstrap replicates. The BI was conducted using parallel MrBayes version 3.2.7a [57]. The ML trees were visualized using ggtree version 2.4.1 [58] and further edited in Adobe Illustrator version 16.0.0.

## 3. Results

### 3.1. Phylogenetic Analyses

**Figure 1:**
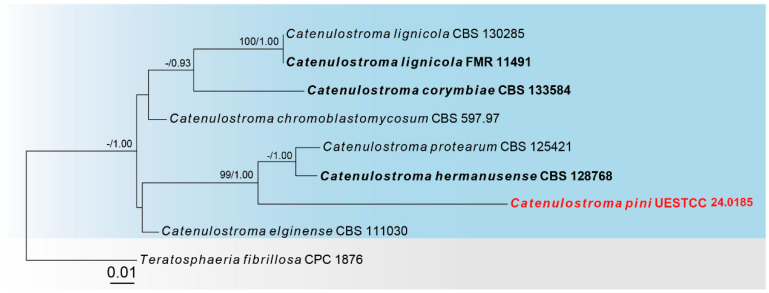
The phylogram of the genus *Catenulostroma* (Teratosphaeriaceae) from ML analysis is based on the concatenated dataset of ITS-LSU-SSU. The tree is rooted with *Teratosphaeria fibrillosa* (CPC 1876). Support values of ML-UFBoot ≥ 95 and Bayesian posterior probabilities ≥ 0.90 were displayed at the nodes as ML/PP. Support values below 95 and 0.90 are indicated by a hyphen (-). Newly collected taxa are shown in red. Strains from type materials are in bold.

Sequences of three loci were successfully obtained for the *Catenulostroma pini* (UESTCC 24.0185). Nine taxa were included in the combined ITS, LSU and SSU sequence data, with *Teratosphaeria fibrillosa* (CPC 1876) as the outgroup (Figure 1). The combined dataset (ITS: 1–644, LSU: 645–1871, SSU: 1872–3619) was composed of 273 distinct patterns, 136 parsimony-informative sites, 324 singleton sites and 3159 constant sites. The best-fit evolution models were K2P + G4 for the ITS partitions, K2P + I for the LSU partition, K2P for the SSU partition. The best-scoring ML tree (lnL = −7762.005) with support values from ML and Bayesian analysis at the node is shown in Figure 1.

According to the multi-locus phylogeny (Figure 1), our collection (UESTCC 24.0185) formed an independent clade sister to *Catenulostroma hermanusense* (CBS 128768) and *C. protearum* (CBS 125421) with 99% ML, 1.00 PP statistical support. Combining the morphological evidence with phylogeny, we propose a new species, *C. pini*, isolated from *Pinus massoniana*.

**Figure 2:**
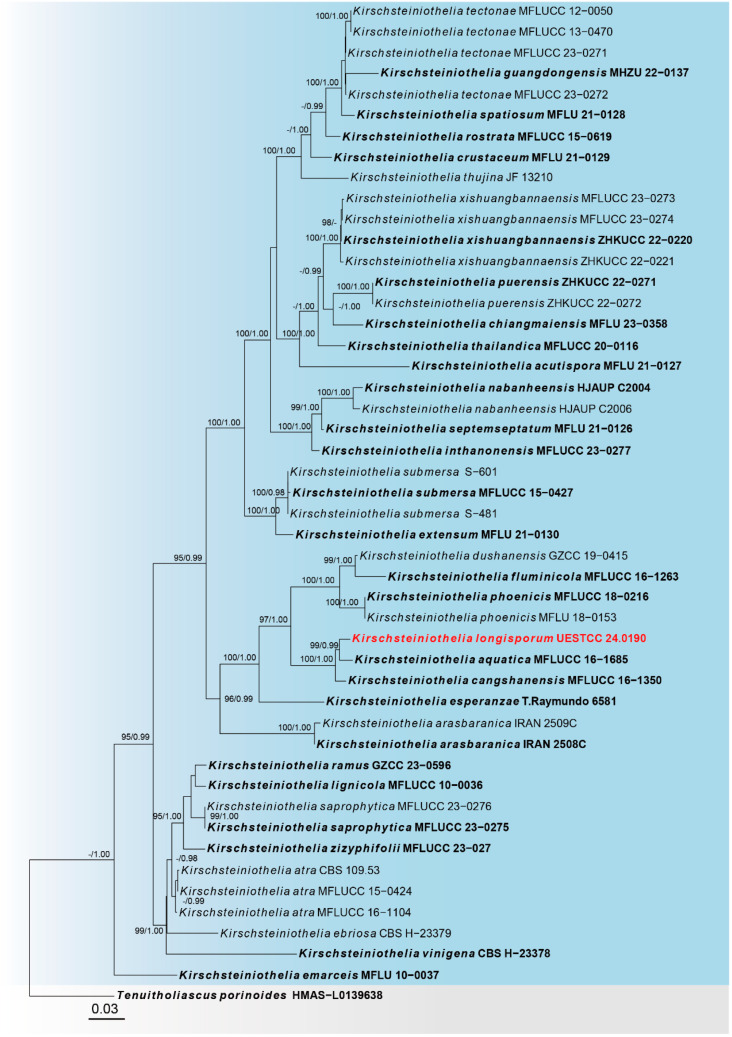
The phylogram of the genus *Kirschsteiniothelia* (Kirschsteiniotheliaceae) from ML analysis is based on the concatenated dataset of ITS-LSU-SSU. The tree is rooted with *Tenuitholiascus porinoides* (HMAS-L0139638). Support values of ML-UFBoot ≥ 95 and Bayesian posterior probabilities ≥ 0.95 were displayed at the nodes as ML/PP. Support values below 95 and 0.95 are indicated by a hyphen (-). Newly collected taxa are shown in red. Strains from type materials are in bold.

Sequences of three loci were successfully obtained for the *Kirschsteiniothelia longisporum* (UESTCC 24.0190). A phylogenetic tree of species in *Kirschsteiniothelia* was constructed (Figure 2), including 48 taxa, with *Tenuitholiascus porinoides* (HMAS-L0139638) as the outgroup. The combined dataset (ITS: 1–507, LSU: 508–1368, SSU: 1369–2392) was composed of 988 distinct patterns, 585 parsimony-informative sites, 252 singleton sites and 1555 constant sites. The best-fit evolution models were GTR + F + G4 for the ITS partitions, GTR + F + G4 for the LSU partition, and K2P + I + G4 for the SSU partition. The best-scoring ML tree (lnL = −14,653.774) with support values from ML and Bayesian analysis at the node is shown in Figure 2.

According to the multi-locus phylogeny (Figure 2), our collection (UESTCC 24.0190) formed a branch sister to *Kirschsteiniothelia aquatica* (MFLUCC 16–1685). Based on the morphological evidence and phylogeny, we propose a new species, *K. longisporum*, isolated from *Pinus taeda*.

**Figure 3:**
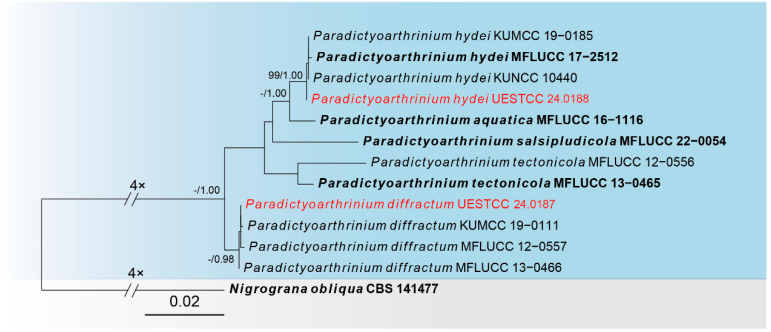
The phylogram of the genus *Paradictyoarthrinium* (Paradictyoarthriniaceae) from ML analysis is based on the concatenated dataset of ITS-LSU-*RPB2*. The tree is rooted with *Nigrograna obliqua* (CBS 141477). Support values of ML-UFBoot ≥ 95 and Bayesian posterior probabilities ≥ 0.95 were displayed at the nodes as ML/PP. Support values below 95 and 0.95 are indicated by a hyphen (-). Newly collected taxa are shown in red. Strains from type materials are in bold.

Sequences of three loci were successfully obtained for the *Paradictyoarthrinium diffractum* (UESTCC 24.0187) and *Paradictyoarthrinium hydei* (UESTCC 24.0188). A phylogenetic tree of species in *Paradictyoarthrinium* was constructed (Figure 3), including 13 taxa, with *Nigrograna obliqua* (CBS 141477) as the outgroup. The combined dataset (ITS: 1–518, LSU: 519–1364, SSU: 1365–2418) was composed of 314 distinct patterns, 73 parsimony-informative sites, 347 singleton sites and 1998 constant sites. The best-fit evolution models were K2P + I for the ITS partitions, K2P + I for the LSU partition, and K2P + I for the *RPB2* partition. The best-scoring ML tree (lnL = −5397.698) with support values from ML and Bayesian analysis at the node is shown in Figure 3.

According to the multi-locus phylogeny (Figure 3), our collection (UESTCC 24.0188) nest with *P. hydei* strains with 99% ML, 1.00 PP statistical support and our collection (UESTCC 24.0187) nest with *P. diffractum* strains. Based on the morphological evidence and phylogeny, we report our collections (UESTCC 24.0188 and UESTCC 24.0187) as new host records of *P. hydei* and *P. diffractum* from *Pinus* spp.

**Figure 4:**
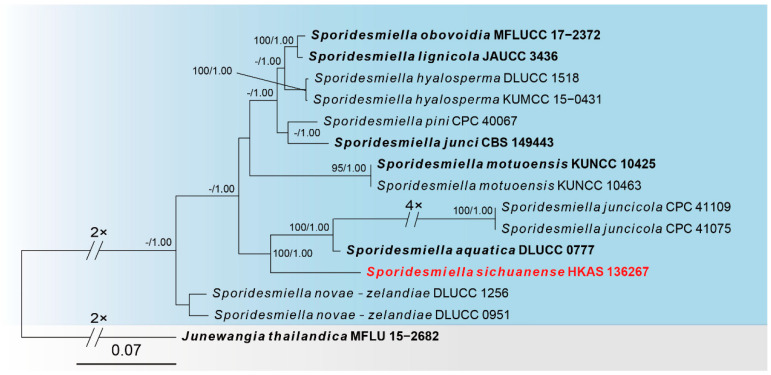
The phylogram of the genus *Sporidesmiella* (Junewangiaceae) from ML analysis is based on the concatenated dataset of ITS-LSU-*RPB2*-*TEF1*. The tree is rooted with *Junewangia thailandica* (MFLU 15–2682). Support values of ML-UFBoot ≥ 95 and Bayesian posterior probabilities ≥ 0.95 were displayed at the nodes as ML/PP. Support values below 95 and 0.95 are indicated by a hyphen (-). Newly collected taxa are shown in red. Strains from type materials are in bold.

Sequences of four loci were successfully obtained for the *Sporidesmiella sichuanensis* (HKAS 136267). A phylogenetic tree of species in *Sporidesmiella* within the Junewangiaceae was constructed (Figure 4), including 15 taxa, with *Junewangia thailandica* (MFLU 15–2682) as the outgroup. The combined dataset (ITS: 1–520, LSU: 521–1326, *RPB2*: 1327–2323, *TEF1*: 2324–3181) was composed of 576 distinct patterns, 375 parsimony-informative sites, 398 singleton sites and 2408 constant sites. The best-fit evolution models were GTR + F + G4 for the ITS partitions, K2P + I for the LSU partition, HKY + F + I for the *RPB2* partition, GTR + F + G4 for the *TEF1* partition. The best-scoring ML tree (lnL = −9362.397) with support values from ML and Bayesian analysis at the node is shown in Figure 4.

According to the multi-locus phylogeny (Figure 4), our collection (HKAS 136267) formed an independent clade sister to *Sporidesmiella aquatica* (DLUCC 0777) and *Sporidesmiella juncicola* strains. Based on the morphological evidence and phylogeny, we identified *S. sichuanensis* as a novel species from *Pinus taeda*.

### 3.2. Taxonomy

**Figure 5:**
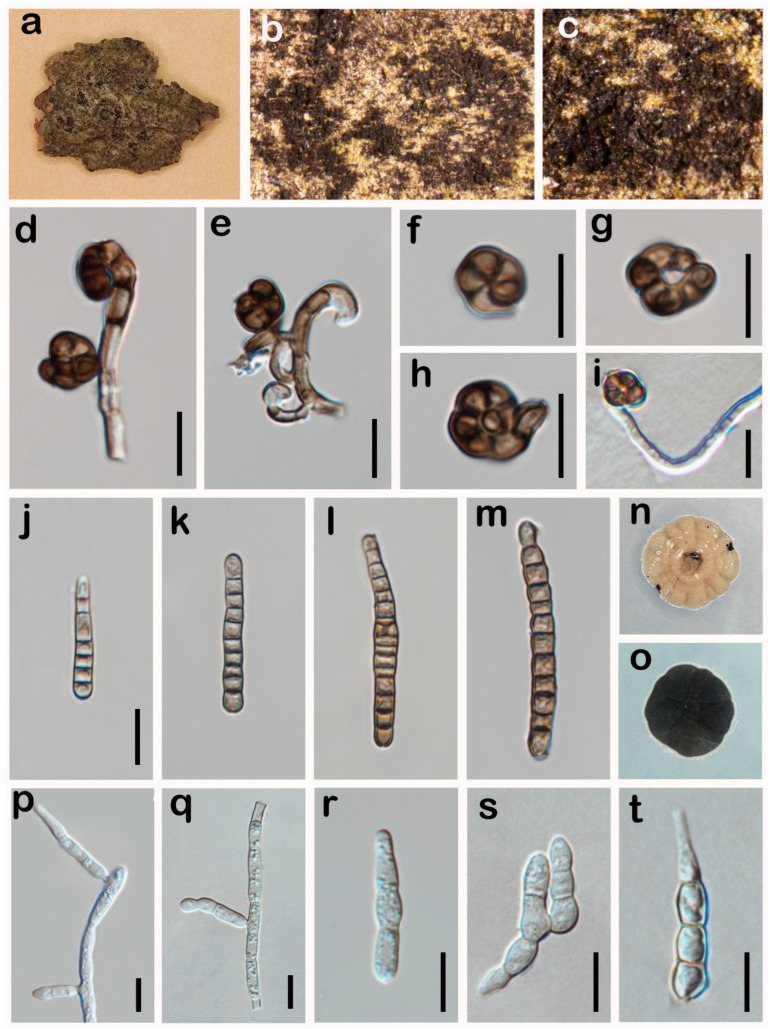
*Catenulostroma pini* (UESTCC 24.0185, holotype). (**a**–**c**) Colonies on the natural substrate; (**d**,**e**) Conidiophores, conidiogenous cells and helicoconidia; (**f**–**h**) Helicoconidia; (**i**) Germinating helicoconidia; (**j**–**m**) Scolecoconidia; (**n**,**o**) Culture characteristics on PDA after 40 days (forth and reverse); (**p**,**q**) Conidiophores, conidiogenous cells and phragmoconidia on PDA; (**r**–**t**) Phragmoconidia on PDA. Scale bars: 10 μm (**d**–**m**,**p**–**t**); Scale bar (**j**) applies to (**j**–**m**).

***Catenulostroma pini*** W.H. Tian & Maharachch., sp. nov. (Figure 5).

*MycoBank*: MB 854981

*Etymology*: Named after the host genus where the fungus was collected.

*Saprobic* on dead bark of *Pinus massoniana* in terrestrial habitats. **Asexual morph:** Hyphomycetes. *Colonies* on the natural substratum effuse, scattered, gregarious, superficial, dark brown, powdery. *Conidiophores* mononematous, micronematous, branched, flexuous, septate, cylindrical, smooth, brown, reduced to conidiogenous cells. *Conidiogenous cells* 1.4–3.3 μm ($\bar{X}$ = 2.2 μm, n = 20) width, integrated, holoblastic–thalloblastic, terminal or conidiophores reduced to conidiogenous cells, cylindrical, brown. *Conidia* three types: *Helicoconidia* 7–10 μm ($\bar{X}$ = 8.5 μm, n = 35) diam., helicospores, solitary, acrogenous, terminal, euseptate, constricted at the septate, circinate, spherical, rounded at apex, thick-walled, brown to dark brown, verruculose. *Scolecoconidia* occasionally formed, 20–42 × 2.6–4.2 μm ($\bar{X}$ = 30 × 3.4 μm, n = 25), occurring in branched chains, scolecospores, catenate, 5–15-septate, cylindrical, straight to slightly curved, pale brown to brown, smooth to finely verruculose. *Phragmoconidia* 12.5–44 × 3–5.5 μm ($\bar{X}$ = 25 × 4 μm, n = 20), in simple or branched chains, cylindric–obclavate, straight to flexuous, septate, constricted at the septate, thick-walled, hyaline to pale brown, verruculose. **Sexual morph:** Undetermined.

*Culture characteristics*: Colonies on PDA reaching 25 mm diam. after 40 days at 25 °C, slow growing, colonies from above: irregularly circular, gray-brown, submerged margins, erumpent, with folded surface, and unevenly lobed; reverse: black.

*Material examined*: CHINA: Sichuan Province, Luzhou City, Daolingou, 29°15′1″ N, 105°42′1″ E, elevation 405 m, 31 March 2023, within dead bark of *Pinus massoniana*, W.H. Tian DLG24 (HKAS 136266, **holotype**), ex-type culture permanently preserved in a metabolically inactive state, UESTCC 24.0185.

*Notes*: Multi-locus phylogenetic analysis indicated that our isolate (UESTCC 24.0185) constitutes an independent clade sister to *Catenulostroma hermanusense* (CBS 128768) and *Catenulostroma protearum* (CBS 125421) (Figure 1). In the NCBI BLASTn search, comparing the ITS and LSU sequence of our isolate (UESTCC 24.0185) and *C. hermanusense* (CBS 128768) revealed 92.42% (549/594 bp, gaps: 17/594 bp), 96.65% (663/686 bp, without gaps) similarity, respectively. Comparing the ITS and LSU sequence of our isolate (UESTCC 24.0185) and *C. protearum* (CBS 125421) revealed 90.82% (475/523 bp, gaps: 17/513 bp), 96.35% (659/684 bp, without gaps) similarity, respectively. Morphologically, our isolate (UESTCC 24.0185) differs from *C. hermanusense* (CBS 128768) by the shape of conidia (helicoconidia, scolecoconidia, phragmoconidia vs. subcylindrical to ellipsoid conidia) and longer and narrower conidia (12.5–44 × 3–5.5 μm vs. 10–25 × 5–10 μm) [59], our isolate (UESTCC 24.0185) differs from *C. protearum* by the shape of conidia (helicoconidia, scolecoconidia, phragmoconidia vs. variable muriform to transversely septate conidia) [60] and relatively narrower conidia (7–10 μm vs. 7–25 μm) [15]. Therefore, based on morphological characteristics and phylogenetic analysis results, we identified *C. pini* as a novel species from *Pinus massoniana* in China.

**Figure 6:**
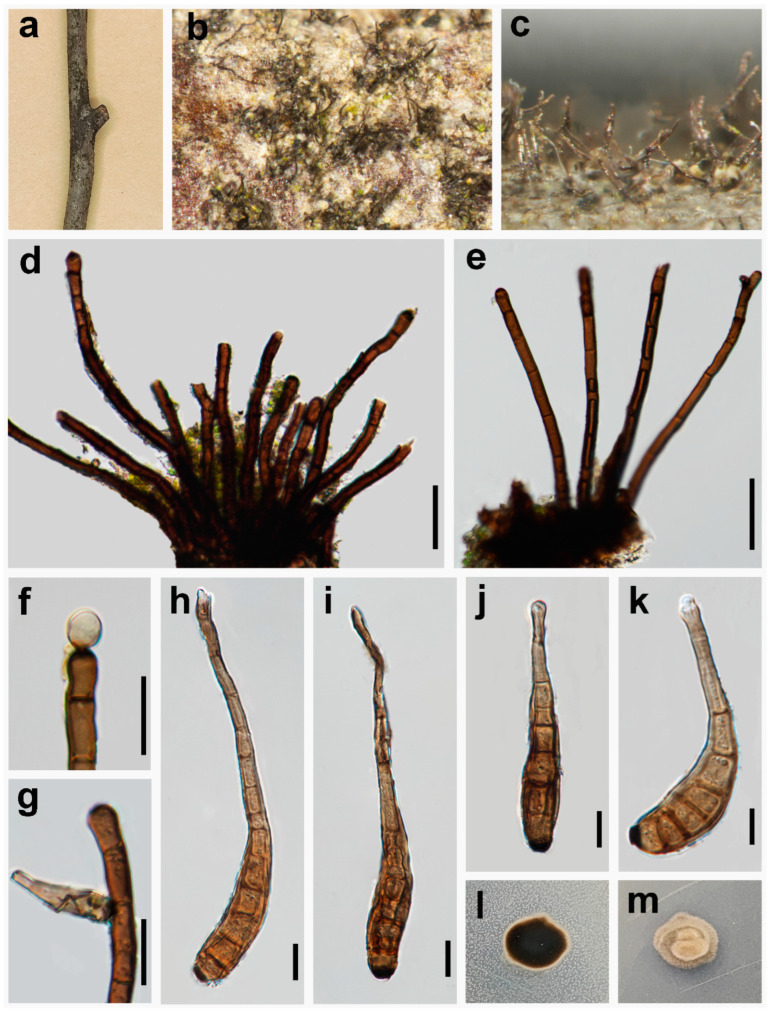
*Kirschsteiniothelia longisporum* (UESTCC 24.0190, holotype). (**a**–**c**) Colonies on the natural substrate; (**d**,**e**) Fascicle and conidiophores; (**f**) Conidiophores with conidiogenous cell and apical conidia; (**g**) Conidiophores with conidiogenous cell and lateral conidia (**h**–**k**) Conidia; (**l**,**m**) Culture characteristics on PDA after 8 days (reverse and forth). Scale bars: 20 μm (**d**–**g**); 10 μm (**h**–**k**).

***Kirschsteiniothelia longisporum*** W.H. Tian & Maharachch., sp. nov. (Figure 6).

*MycoBank*: MB 854982

*Etymology*: The epithet refers to the long spores.

*Saprobic* on a dead branch of *Pinus taeda* in terrestrial habitats. **Asexual morph:** Hyphomycetes. *Colonies* on the natural substratum effuse, hairy, black. *Mycelium* superficial, hairy, scattered, dark brown to black. *Conidiophores* 115–285 × 6.5–14 μm ($\bar{X}$ = 215 × 9 μm, n = 30), macronematous, mononematous, sometimes branched, solitary or fasciculate, erect, straight or slightly flexuous, cylindrical, septate, verruculose, dark brown to black. *Conidiogenous cells* holoblastic, integrated, terminal and intercalary, cylindrical, obtuse at apex, dark brown, verruculose. *Conidia* 35–130 × 8.5–15 μm ($\bar{X}$ = 65 × 11 μm, n = 35), tapering to 2–4.5 μm ($\bar{X}$ = 3, n = 35) at the distal end, with a blackish-brown 3–7 μm wide ($\bar{X}$ = 4.5, n = 35) scar at the base, phragmoconidia, solitary, acrogenous, 3–15-distoseptate, cylindric–obclavate, elongated, wide at the middle and lower part, straight or flexuous, uneven width, slender and rounded at apex, truncate at base, brown, thick-walled, verruculose, secession schizolytic. **Sexual morph:** Undetermined.

Culture characteristics—Colony on PDA reaching 11 mm diam. in 8 days at 25 °C in the dark, colonies from above: irregular circular, grey, uneven entire, raised in centre, with denser mycelium at the centre; reverse: black, cream at the margin, margin undulated.

*Material examined*: CHINA: Sichuan Province, Chengdu City, Jiudaoguai, 30°30′21″ N, 103°53′47″ E, elevation 502 m, 19 October 2023, within dead branches of *Pinus taeda*, W.H. Tian JDG36 (HKAS 136268, **holotype**), ex-type culture permanently preserved in a metabolically inactive state, CGMCC 3.27599 = UESTCC 24.0190.

*Notes*: Phylogenetic analysis based on the combined dataset of ITS, LSU and SSU loci revealed that our collection (UESTCC 24.0190) forms a branch sister to *Kirschsteiniothelia aquatica* (MFLUCC 16–1685) and *K. cangshanensis* (MFLUCC 16–1350) (Figure 2). Comparing the ITS sequence of our collection (UESTCC 24.0190) with *K. aquatica* (MFLUCC 16–1685) revealed 94.67% (515/544 bp, gaps: 4/544 bp) similarity, and with *k. cangshanensis* (MFLUCC 16–1350) revealed 91.91% (477/519 bp, gaps: 4/519 bp) similarity. Morphologically, our collection differs from the *K. aquatica* (MFLUCC 16–1685) and *K. cangshanensis* (MFLUCC 16–1350) by larger conidiophores (115–285 × 6.5–14 μm vs. 114–151 × 7–8 μm vs. 105–135 × 6–8 μm) and bigger conidia (35–130 × 8.5–15 μm vs. 35–46 × 7.5–8.8 μm vs. 33–43 × 7.5–8.5 μm) [61]. Our collection (UESTCC 24.0190) shares similar characteristics with *K. fluminicola* (MFLUCC 16–1263) in having slender conidia rounded at apex and multi-septate at maturity [61]. However, the conidiophores of our collection (UESTCC 24.0190) are wider than *K. fluminicola* (MFLUCC 16–1263) (6.5–14 μm vs. 7–9 μm), and the conidia of our collection (UESTCC 24.0190) is relatively bigger than *K. fluminicola* (MFLUCC 16–1263) (35–130 × 8.5–15 μm vs. 47.5–86.5 × 8–10 μm) [61]. Thus, our collection UESTCC 24.0190 is described as a new species based on morphological observation and phylogenetic evidence.

**Figure 7:**
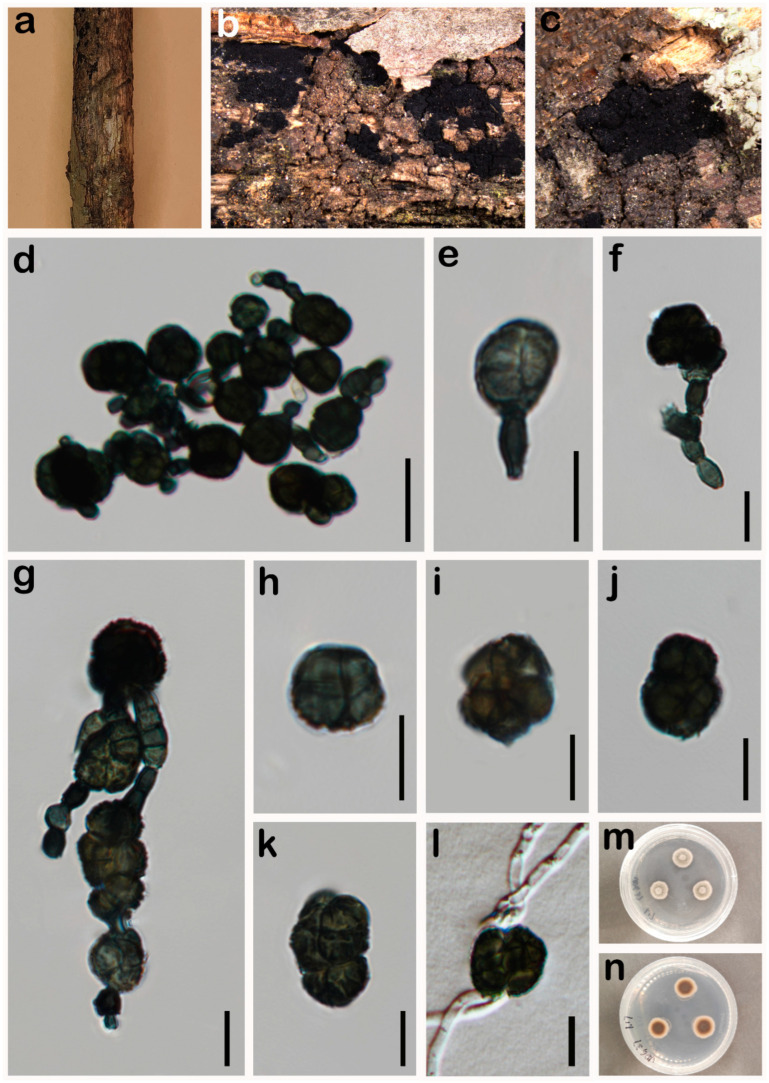
*Paradictyoarthrinium diffractum* (UESTCC 24.0187). (**a**–**c**) Colonies on the natural substrate; (**d**–**g**) Conidiophores, conidiogenous cells and conidia; (**h**–**k**) Conidia; (**l**) Germinating conidium; (**m**,**n**) Culture characteristics on PDA after 10 days (forth and reverse). Scale bars: 20 μm (**d**); 10 μm (**e**–**l**).

***Paradictyoarthrinium diffractum*** Matsush., Matsush. Mycol. Mem. 9: 18 (1996) (Figure 7).

*Saprobic* on a dead branch of *Pinus taeda* in terrestrial habitats. **Asexual morph:** Hyphomycetes. *Colonies* on the natural substrate scattered, gregarious, superficial, black, powdery. *Conidiophores* thick-walled, black, macronematous, sometimes micronematous, erect to slightly curved, short, branched or unbranched, arising from hyphae, slightly constricted at the septa. *Conidiogenous cells* 3.8–8.3 × 2.5–7 μm ($\bar{X}$ = 5.3 × 4 μm, n = 25), blastic, mostly terminal, determinate, peacock green. *Conidia* 14–28 × 10–20 μm ($\bar{X}$ = 20 × 15 μm, n = 35), dictyoconidia, solitary or formed in chains, unevenly dictyoseptate, subglobose to ellipsoidal, verrucose, dark green when immature and dark when mature. **Sexual morph:** Undetermined.

*Culture characteristics*: Colonies on PDA reaching 20 mm diam. after 10 days at 25 °C, colonies from above: grey at the centre, creamy white until margin, hyaline mycelia at the entire edge, dense, fluffy, and circular, reverse: dark-olivaceous brown at the centre, and creamy white towards the edge.

*Material examined*: China, Sichuan Province, Chengdu City, Jiudaoguai, 30°30′21″ N, 103°53′47″ E, elevation 502 m, 19 October 2023, within dead branches of *Pinus taeda*, W.H. Tian JDG37 (HUEST 24.0204), living culture permanently preserved in a metabolically inactive state, UESTCC 24.0187.

*Notes*: According to the multi-locus phylogeny, our collection (UESTCC 24.0187) is nested with *P. diffractum* strains (Figure 3). Based on the BLASTn NCBI GenBank database search of ITS, LSU and *RPB2* sequences, our collection (UESTCC 24.0187) is 99% similar to *P. diffractum*. In addition, the morphological characteristics of our collection (UESTCC 24.0187) overlap with the *P. diffractum* in having powdery colonies, lacking conidiophores, and solitary or formed in chains, pleomorphic conidia [62]. Thus, based on morphological comparison and phylogenetic analyses, we report our collections (UESTCC 24.0187) as a new host record of *P. diffractum* from *Pinus taeda*.

**Figure 8:**
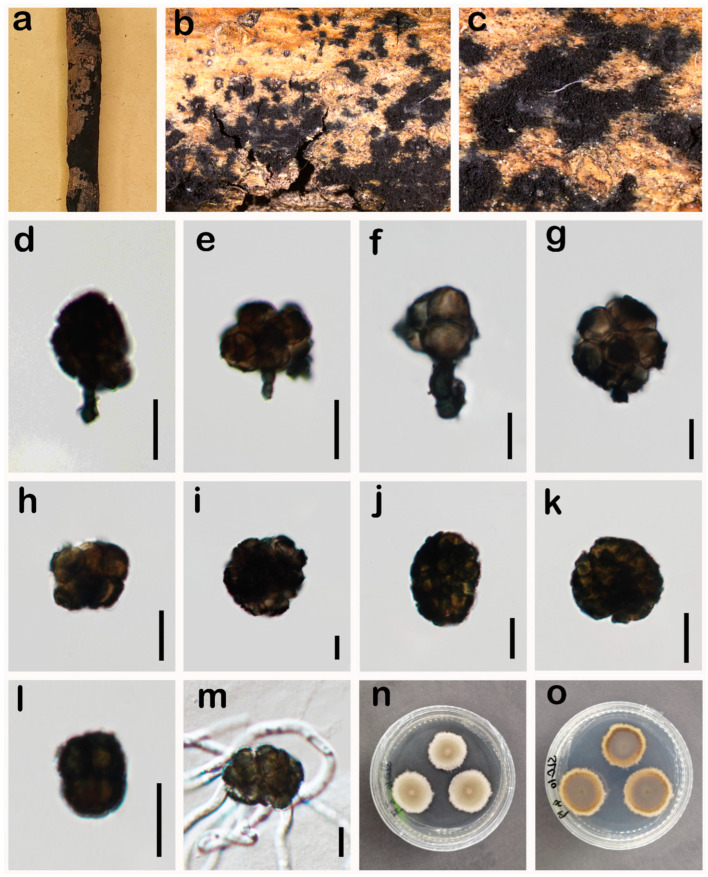
*Paradictyoarthrinium hydei* (UESTCC 24.0188). (**a**–**c**) Colonies on the natural substrate; (**d**–**g**) Conidiogenous cells and conidia; (**h**–**l**) Conidia; (**m**) Germinating conidium; (**n**,**o**) Culture characteristics on PDA after 20 days (forth and reverse). Scale bars: 10 μm (**d**–**m**).

***Paradictyoarthrinium hydei*** N.G. Liu & J.K. Liu, in Liu, Luo, Liu, Cheewangkoon & Chaiwat, Phytotaxa 338(3): 290 (2018) (Figure 8).

*Saprobic* on a dead branch of *Pinus* sp. in terrestrial habitats. **Asexual morph:** Hyphomycetes. *Colonies* on the natural substrate, scattered, gregarious, superficial, black, powdery. *Conidiophores* macronematous, rarely micronematous, thick-walled, black, erect to slightly curved, short, branched or unbranched, arising from hyphae, slightly constricted at the septa. *Conidiogenous cells* 4.3–5.8 × 2.7–5 μm ($\bar{X}$ = 5 × 4 μm, n = 25), monoblastic, terminal, determinate, integrated, dark green. *Conidia* 18–40 × 9–35 μm ($\bar{X}$ = 28 × 22 μm, n = 35), dictyoconidia, solitary or formed in chains, unevenly dictyoseptate, subglobose to ellipsoidal, constricted at the septa, verrucose, dark green when immature and dark when mature. **Sexual morph:** Undetermined.

*Culture characteristics*: Colonies on PDA reaching 34 mm diam. after 20 days at 25 °C, colonies from above: circular, divergent at the margin, dense, slightly raised, white at the margin, grey at the centre, reverse: dark-olivaceous brown at the centre, and creamy white towards the edge.

*Material examined*: China, Sichuan Province, Neijiang City, Songlin Village, 29°32′19″ N, 105°9′28″ E, elevation 373 m, 1 April 2023, decaying branches of *Pinus* sp., W.H. Tian SLC10 (HUEST 24.0205), living culture permanently preserved in a metabolically inactive state, UESTCC 24.0188.

*Notes*: Multi-locus phylogeny indicates that our collection’s (UESTCC 24.0188) sister is the *Paradictyoarthrinium hydei* group with 99% ML, 1.00 PP statistical support (Figure 3). In the NCBI BLASTn search, the ITS, LSU and *RPB2* sequences of our collection (UESTCC 24.0188) are 99% similar to *P. hydei.* In addition, as morphological characteristics examined largely overlapped with the type strain of *P. hydei* [29], we report our collections (UESTCC 24.0188) as a new host record of *P. hydei* from *Pinus* sp.

**Figure 9:**
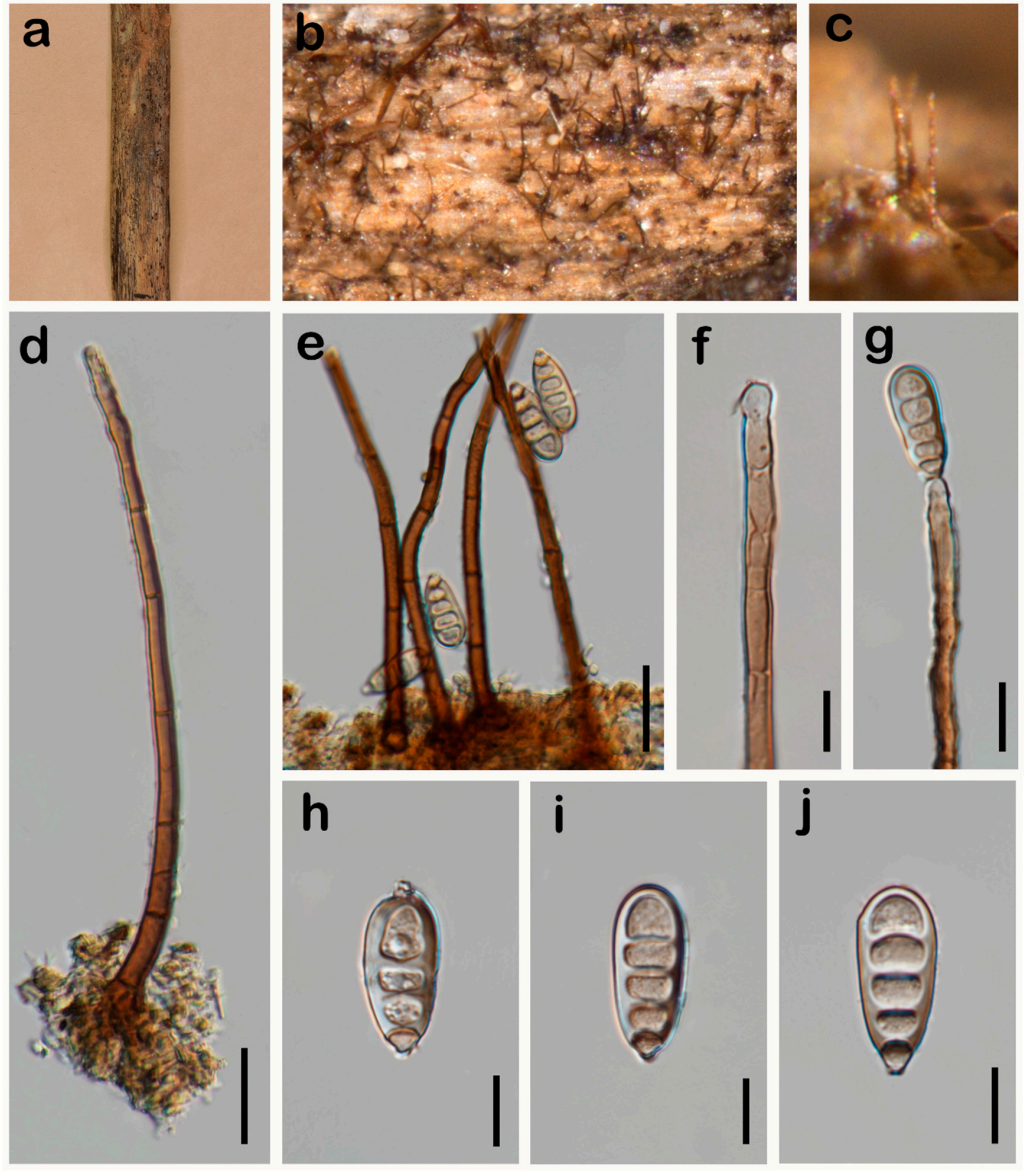
*Sporidesmiella sichuanensis* (HKAS 136267, holotype). (**a**–**c**) Colonies on the natural substrate; (**d**) Conidiophores; (**e**) Conidiophores and conidia; (**f**,**g**) Conidiophores with conidiogenous cell and apical conidia; (**h**–**j**) Conidia. Scale bars: 20 μm (**d**,**e**); 10 μm (**f**–**j**).

***Sporidesmiella sichuanensis*** W.H. Tian & Maharachch., sp. nov. (Figure 9).

*MycoBank*: MB 854983

*Etymology*: Named after the Sichuan province, China, where the holotype was collected.

*Saprobic* on a dead branch of *Pinus taeda* in terrestrial habitats. **Asexual morph:** Hyphomycetes. *Colonies* on the natural substratum effuse, hairy, black. *Mycelium* superficial, hairy, yellow-brown to brown, scattered. *Conidiophores* 108–178 × 3.2–5.7 μm ($\bar{X}$ = 137 × 4 μm, n = 20), macronematous, mononematous, solitary, unbranched, erect, straight or slightly flexuous, cylindrical, 6–12-septate, smooth, yellow-brown, paler towards the apex. *Conidiogenous cells* holoblastic, polyblastic, integrated, sympodial, terminal, cylindrical, subhyaline to pale brown, smooth, percurrently extended, with the extensions towards the apex. *Conidia* 20–27 × 9.5–12 μm ($\bar{X}$ = 24 × 10 μm, n = 35), phragmoconidia, solitary, acrogenous, 3–4-distoseptate, cylindric–obclavate, widened and rounded at the apex, uneven width, truncate at the base, subhyaline to pale brown, thick-walled, smooth. **Sexual morph:** Undetermined.

*Material examined*: CHINA: Sichuan Province, Chengdu City, Jiudaoguai, 30°30′21″ N, 103°53′47″ E, elevation 502 m, 19 October 2023, within dead branches of *Pinus taeda*, W.H. Tian JDG40_1 (HKAS 136267, **holotype**).

*Notes*: Phylogenetic analyses of combined ITS, LSU, *RPB2* and *TEF1* sequence data showed that our collection (HKAS 136267) forms a separate branch sister to *Sporidesmiella aquatica* and *Sporidesmiella juncicola* (Figure 4). The closest match to our collection (HKAS 136267) is *S. aquatica* (DLUCC 0777) (Figure 4). Sequence comparison for the ITS, LSU and *TEF1* region between our collection (HKAS 136267) and the type strain of *S. aquatica* showed 87.74% (315/359 bp, gaps: 14/359 bp), 96.95% (699/721 bp, gaps: 5/721 bp) and 95.25% (802/842 bp, without gaps) base pair similarity. However, morphologically, our collection (HKAS 136267) differs from the *S. aquatica* (DLUCC 0777) by smaller conidiophores (108–178 × 3.2–5.7 μm vs. 178–228 × 8–10 μm) and smaller conidia (20–27 × 9.5–12 μm vs. 51–59 × 18–22 μm) [10]. Therefore, based on morphology and phylogenetic analyses, we introduce *S. sichuanensis* as a novel species from *Pinus taeda* in China.

## 4. Discussion

In this study, two rarely seen species from *Pinus* spp. were reported. *Catenulostroma pini* sp. nov., isolated from the dead bark of *Pinus massoniana*, is the seventh species of the genus and the first time that the genus *Catenulostroma* has been recorded in China [15,16,59]. *Sporidesmiella sichuanensis* sp. nov., isolated from a dead branch of *Pinus taeda*, is the eleventh species in the genus to have sequence data. *Sporidesmiella* is an old genus with origins dating back to the 18th century (previously as *Sporidesmium*); hence, molecular data are unavailable for most species [32]. *Sporidesmiella pini*, was isolated from needles of *Pinus sylvestris* in the Netherlands [63]. This suggests that many unique and rare taxa on *Pinus* spp. may still await discovery and exploration.

*Catenulostroma pini* sp. nov. is an interesting species in which three conidial morphologies were observed. The first type is a helicoconidia small terminal conidia, characterized by helicospores that are solitary, acrogenous, euseptate, constricted at the septa, circinate, and spherical. This morphology is slightly similar to that of *C. protearum* obtained from OA, as reported by Crous et al. [60]. However, the conidia in our collection are circinate and spherical, whereas the conidia of *C. protearum* are variably muriform to transversely septate [60]. The transversely septate conidia of *C. protearum* are very similar to the second conidial morphology in our collection [60]. They all occur in branched chains, forming chain-like, septate, and scolecospores, which are also similar to the conidia of *C. chromoblastomycosum* [15,60]. After germination and sporulation of helicoconidia on PDA, we observed a third morphology of conidia characterized by simple or branched chains, cylindric–obclavate, straight to flexuous, hyaline to pale brown, septate, constricted at the septate. Similar to *C. hermanusense*, conidia are in simple or branched chains but with different morphologies: cylindric–obclavate and constricted at the septa, compared to subcylindrical to ellipsoid [59]. Besides the unique and diverse morphology of the genus *Catenulostroma*, it is noteworthy that *C. chromoblastomycosum* was described as a case of human chromoblastomycosis [15]. Currently, no other species of this genus have been found capable of infecting humans and causing disease.

In this study, four hyphomycetes fungi collected from *Pinus* spp. were isolated and identified: *Catenulostroma pini* sp. nov. (Teratosphaeriaceae), *Kirschsteiniothelia longisporum* sp. nov. (Kirschsteiniotheliaceae), *Paradictyoarthrinium diffractum* and *P. hydei* of Paradictyoarthriniaceae, all within Dothideomycetes. Dothideomycetes represent the largest and most diverse class of ascomycete fungi [6,25,64,65]. This class includes over 25 orders, 110 families and more than 19,000 species [66]. Their representatives have an incredibly diverse lifestyle and can be associated with various hosts and substrates [6]. Another collection was identified as *Sporidesmiella sichuanensis* sp. nov., a saprophyte in the Junewangiaceae family within Sordariomycetes. *Sporidesmiella* is a polyphyletic genus. Based on phylogenetic analysis of the LSU and *RPB2* datasets, Shenoy et al. [67] classified *S. fusiformis* into the family Melanommataceae within Dothideomycetes. Subsequently, using combined ITS, LSU, *RPB2*, and *TEF1* sequence data, many species in the genus were reassigned to Junewangiaceae within Sordariomycetes [10,33,34,35,36]. The diversity of fungal groups in Sordariomycetes is high, and most species are saprophytic fungi that can degrade organic matter in nature and promote the material cycle of the ecosystem [68,69,70].

## Figures and Tables

**Table 1:** Loci used in this study with the corresponding PCR primers and conditions.

Locus	PCR Primers	PCR: Thermal Cycles	References
ITS	ITS9mun or ITS5/ ITS4_KYO1 or ITS4	(94 °C: 30 s, 56 °C: 30 s, 72 °C: 30 s) × 35 cycles	[44,45]
LSU	LR0R/LR5	(94 °C: 30 s, 56 °C: 30 s, 72 °C: 1 min) × 35 cycles	[46,47]
SSU	PNS1/ NS41	(94 °C: 30 s, 56 °C: 30 s, 72 °C: 1 min) × 35 cycles	[48]
*TEF1*	EF1-983/ EF1-2218R	(94 °C: 30 s, 58 °C: 30 s, 72 °C: 1 min) × 35 cycles	[49,50]
*RPB2*	dRPB2-5f/dRPB2-7r	(94 °C: 30 s, 58 °C: 30 s, 72 °C: 1 min) × 35 cycles	[51]

**Table 2:** Species details and their GenBank accession numbers used in phylogenetic analyses of *Catenulostroma*. Type strains are in bold, and newly generated sequences are in red.

Species	Culture/Specimen No.	GenBank Accession Numbers		
		SSU	LSU	ITS
*Catenulostroma chromoblastomycosum*	CBS 597.97	GU214516	EU019251	AJ244260
***C. corymbiae***	**CBS 133584**	**–**	**KC005805**	**KC005783**
*C. elginense*	CBS 111030	GU214517	EU019252	–
***C. hermanusense***	**CBS 128768**	**–**	**JF499853**	**JF499833**
*C. lignicola*	CBS 130285	–	NG_059023	NR_154848
***C. lignicola***	**FMR 11491**	**–**	**KY853489**	**KY853429**
***C. pini***	**UESTCC 24.0185**	**PQ046106**	**PQ038269**	**PQ038262**
*C. protearum*	CBS 125421	–	KF902090	MH863677
*Teratosphaeria fibrillosa*	CPC 1876	–	GU214506	EU019282

**Table 3:** Species details and their GenBank accession numbers used in phylogenetic analyses of *Kirschsteiniothelia*. Type strains are in bold, and newly generated sequences are in red.

Species	Culture/Specimen No.	GenBank Accession Numbers		
		SSU	LSU	ITS
***Kirschsteiniothelia acutispora***	**MFLU 21-0127**	**ON980754**	**ON980758**	**OP120780**
***K. aquatica***	**MFLUCC 16-1685**	**MH182618**	**MH182594**	**MH182587**
*K. arasbaranica*	IRAN 2509C	KX621988	KX621987	KX621986
***K. arasbaranica***	**IRAN 2508C**	**KX621985**	**KX621984**	**KX621983**
*K. atra*	CBS 109.53	AY016344	AY016361	–
*K. atra*	MFLUCC 16-1104	MH182615	MH182589	MH182583
*K. atra*	MFLUCC 15-0424	KU500585	KU500578	KU500571
***K. cangshanensis***	**MFLUCC 16-1350**	**–**	**MH182592**	**MH182584**
***K. chiangmaiensis***	**MFLU 23-0358**	**OR575475**	**OR575474**	**OR575473**
***K. crustaceum***	**MFLU 21-0129**	**–**	**MW851854**	**MW851849**
***K. dushanensis***	**GZCC 19-0415**	**MW134610**	**MW133830**	**OP377845**
***K. ebriosa***	**CBS H-23379**	**–**	**LT985885**	**–**
***K. emarceis***	**MFLU 10-0037**	**–**	**NG_059454**	**NR_138375**
***K. extensum***	**MFLU 21-0130**	**–**	**MW851855**	**MW851850**
***K. esperanzae***	**T. Raymundo 6581**	**–**	**OQ880482**	**OQ877253**
***K. fluminicola***	**MFLUCC 16-1263**	**–**	**MH182588**	**MH182582**
***K. guangdongensis***	**MHZU 22-0137**	**–**	**OR164974**	**OR164946**
***K. inthanonensis***	**MFLUCC 23-0277**	**OR764784**	**OR762781**	**OR762773**
***K. longisporum***	**UESTCC 24.0190**	**PQ046108**	**PQ038273**	**PQ038266**
***K. lignicola***	**MFLUCC 10-0036**	**HQ441569**	**HQ441568**	**HQ441567**
*K. nabanheensis*	HJAUP C2006	OQ023037	OQ023275	OQ023274
***K. nabanheensis***	**HJAUP C2004**	**OQ023038**	**OQ023273**	**OQ023197**
***K. ramus***	**GZCC 23-0596**	**–**	**OR091333**	**NR_190260**
*K. phoenicis*	MFLU 18-0153	–	NG_064508	NR_158532
***K. phoenicis***	**MFLUCC 18-0216**	**MG859979**	**MG860484**	**MG859978**
*K. puerensis*	ZHKUCC 22-0272	OP451021	OP451018	OP450978
***K. puerensis***	**ZHKUCC 22-0271**	**OP451020**	**OP451017**	**OP450977**
***K. rostrata***	**MFLUCC 15-0619**	**KY697278**	**KY697276**	**KY697280**
***K. septemseptatum***	**MFLU 21-0126**	**ON980752**	**ON980757**	**OP120779**
***K. saprophytica***	**MFLUCC 23-0275**	**–**	**OR762783**	**OR762774**
*K. saprophytica*	MFLUCC 23-0276	–	OR762782	OR762775
***K. spatiosum***	**MFLU 21-0128**	**ON980753**	**–**	**NR_187065**
*K. submersa*	S-481	MH182616	MH182591	–
*K. submersa*	S-601	–	MH182593	MH182585
***K. submersa***	**MFLUCC 15-0427**	**KU500584**	**KU500577**	**KU500570**
***K. tectonae***	**MFLUCC 12-0050**	**–**	**KU764707**	**KU144916**
*K. tectonae*	MFLUCC 13-0470	–	–	KU144924
*K. tectonae*	MFLUCC 23-0271	OR764782	OR762779	OR762771
*K. tectonae*	MFLUCC 23-0272	OR764783	OR762780	OR762772
***K. thailandica***	**MFLUCC 20-0116**	**MT984280**	**MT984443**	**MT985633**
*K. thujina*	JF13210	KM982717	KM982718	KM982716
***K. vinigena***	**CBS H-23378**	**–**	**NG_075229**	**–**
*K. xishuangbannaensis*	ZHKUCC 22-0221	OP289565	OP303182	OP289563
***K. xishuangbannaensis***	**ZHKUCC 22-0220**	**OP289564**	**OP303181**	**OP289566**
*K. xishuangbannaensis*	MFLUCC 23-0273	OR764781	OR762778	OR762770
*K. xishuangbannaensis*	MFLUCC 23-0274	OR764780	OR762777	OR762769
***K. zizyphifolii***	**MFLUCC 23-0270**	**OR764779**	**OR762776**	**OR762768**
***Tenuitholiascus porinoides***	**HMAS-L0139638**	**MK352441**	**MK206259**	**–**

**Table 4:** Species details and their GenBank accession numbers used in phylogenetic analyses of *Paradictyoarthrinium*. Type strains are in bold, and newly generated sequences are in red.

Species	Culture/Specimen No.	GenBank Accession Numbers		
		LSU	ITS	*RPB2*
***Nigrograna obliqua***	**CBS 141477**	**KX650560**	**KX650560**	**KX650580**
***Paradictyoarthrinium aquatica***	**MFLUCC 16-1116**	**MG747495**	**MG747496**	**MG780231**
*P. diffractum*	MFLUCC 13-0466	KP744498	KP744455	KX437764
*P. diffractum*	MFLUCC 12-0557	KP744497	KP744454	KX437765
*P. diffractum*	KUMCC 19-0111	MN582756	MN582741	MN643158
*P. diffractum*	UESTCC 24.0187	PQ038271	PQ038264	PQ050360
*P. hydei*	KUNCC 10440	OQ146990	OQ135179	–
*P. hydei*	KUMCC 19-0185	–	MN582742	MN643159
***P. hydei***	**MFLUCC 17-2512**	**MG747497**	**MG747498**	**MG780232**
*P. hydei*	UESTCC 24.0188	PQ038272	PQ038265	PQ050361
***P. salsipludicola***	**MFLUCC 22-0054**	**OR589801**	**OR589800**	**–**
***P. tectonicola***	**MFLUCC 13-0465**	**KP744500**	**KP744456**	**KX437763**
*P. tectonicola*	MFLUCC 12-0556	KP744499	–	–

**Table 5:** Species details and their GenBank accession numbers used in phylogenetic analyses of *Sporidesmiella*. Type strains are in bold, and newly generated sequences are in red.

Species	Culture/Specimen No.	GenBank Accession Numbers			
		LSU	ITS	*RPB2*	*TEF1*
***Junewangia thailandica***	**MFLU 15-2682**	**MW287762**	**–**	**–**	**–**
***Sporidesmiella aquatica***	**DLUCC 0777**	**MK849843**	**MK828692**	**–**	**MN194034**
*S. hyalosperma*	DLUCC 1518	MK849842	MK828691	MN124523	MN194033
*S. hyalosperma*	KUMCC 15-0431	MK849841	MK828690	MN124522	MN194032
***S. junci***	**CBS 149443**	**NG_229048**	**OP675893**	**OP676106**	**–**
*S. juncicola*	CPC 41075	OK663757	OK664718	OK651165	–
*S. juncicola*	CPC 41109	OK663758	OK664719	OK651166	OK651188
***S. lignicola***	**JAUCC 3436**	**OK091615**	**MZ613187**	**OK323222**	**OK323223**
***S. motuoensis***	**KUNCC 10425**	**OR229720**	**OP626348**	**–**	**–**
*S. motuoensis*	KUNCC 10463	OR229719	OR286630	–	–
*S. novae-zelandiae*	DLUCC 0951	MK849847	MK828695	MN124526	MN194037
*S. novae-zelandiae*	DLUCC 1256	MK849845	MK828693	MN124525	MN194036
***S. obovoidia***	**MFLUCC 17-2372**	**MW287766**	**MW286492**	**–**	**–**
*S. pini*	CPC 40067	OK663786	OK664747	OK651177	–
***S. sichuanensis***	**H** **KAS** **136267**	**PQ038270**	**PQ038263**	**PQ050359**	**PQ050356**

## Data Availability

The data presented in this study are openly available in NCBI GenBank at https://www.ncbi.nlm.nih.gov/nuccore.

## References

[1] Taylor T.N., Krings M., Taylor E.L., Taylor T.N., Krings M., Taylor E.L. (2015). 11—Fungal spores. Fossil Fungi.

[2] Seifert K.A., Gams W. (2011). The genera of Hyphomycetes—2011 update. Persoonia.

[3] Wijayawardene N.N., Phillips A.J.L., Pereira D.S., Dai D.Q., Aptroot A., Monteiro J.S., Druzhinina I.S., Cai F., Fan X., Selbmann L. (2022). Forecasting the number of species of asexually reproducing fungi (Ascomycota and Basidiomycota). Fungal Divers..

[4] Lombard L., Houbraken J., Decock C., Samson R., Meijer M., Réblová M., Groenewald J.Z., Crous P.W. (2016). Generic hyper-diversity in Stachybotriaceae. Persoonia.

[5] Voglmayr H., Jaklitsch W.M. (2017). *Corynespora*, *Exosporium* and *Helminthosporium* revisited—New species and generic reclassification. Stud. Mycol..

[6] Haridas S., Albert R., Binder M., Bloem J., LaButti K., Salamov A., Andreopoulos B., Baker S.E., Barry K., Bills G. (2020). 101 Dothideomycetes genomes: A test case for predicting lifestyles and emergence of pathogens. Stud. Mycol..

[7] Cannon P.F., Kirk P.M. (2007). *Fungal* *Families of the World*. https://www.cabidigitallibrary.org/doi/book/10.1079/9780851998275.0000.

[8] Dong W., Wang B., Hyde K.D., McKenzie E.H.C., Raja H.A., Tanaka K., Abdel-Wahab M.A., Abdel-Aziz F.A., Doilom M., Phookamsak R. (2020). Freshwater Dothideomycetes. Fungal Divers..

[9] Li W.L., Luo Z.L., Liu J.K., Bhat D.J., Bao D.F., Su H.Y., Hyde K.D. (2017). Lignicolous freshwater fungi from China I: *Aquadictyospora lignicola* gen. et sp. nov. and new record of *Pseudodictyosporium wauense* from northwestern Yunnan Province. Mycosphere.

[10] Luo Z.L., Hyde K.D., Liu J.K., Maharachchikumbura S.S.N., Jeewon R., Bao D.F., Bhat D.J., Lin C.G., Li W.L., Yang J. (2019). Freshwater Sordariomycetes. Fungal Divers..

[11] Hu Y.F., Liu J., Zhang X.G., Castañeda-Ruiz R., Ma J. (2023). *Acropleurophialis simplex* gen. & sp. nov. from China. Mycotaxon.

[12] Liu J., Hu Y.F., Cui R.Q., Castañeda-Ruiz R., Xu Z.H., Ma J. (2022). New species of *Catenularia* and *Fuscocatenula* from Xishuangbanna, China. Mycotaxon.

[13] Liu J., Hu Y.F., Zhang K., Zhang X.G., Castañeda-Ruiz R., Ma J. (2022). *Zasmidium sinense* sp. nov. from Guangdong, China. Mycotaxon.

[14] Schell W.A. (2002). Dematiaceous hyphomycetes. Pathogenic Fungi in Humans and Animals.

[15] Crous P.W., Braun U., Groenewald J.Z. (2007). Mycosphaerella is polyphyletic. Stud. Mycol..

[16] Hernández-Restrepo M., Gené J., Castañeda-Ruiz R.F., Mena-Portales J., Crous P.W., Guarro J. (2017). Phylogeny of saprobic microfungi from Southern Europe. Stud. Mycol..

[17] Hawksworth D.L. (1985). *Kirschsteiniothelia*, a new genus for the *Microthelia incrustans*-group (Dothideales). Bot. J. Linn. Soc..

[18] Sun Y., Jayawardena R.S., Hyde K.D., Wang Y. (2021). *Kirschsteiniothelia thailandica* sp. nov. (Kirschsteiniotheliaceae) from Thailand. Phytotaxa.

[19] Schoch C.L., Crous P.W., Groenewald J.Z., Boehm E.W.A., Burgess T.I., de Gruyter J., de Hoog G.S., Dixon L.J., Grube M., Gueidan C. (2009). A class-wide phylogenetic assessment of Dothideomycetes. Stud. Mycol..

[20] Boonmee S., Ko T.W., Chukeatirote E., Hyde K.D., Chen H., Cai L., McKenzie E.H., Jones E.B., Kodsueb R., Hassan B.A. (2012). Two new *Kirschsteiniothelia* species with *Dendryphiopsis* anamorphs cluster in Kirschsteiniotheliaceae fam. nov. Mycologia.

[21] Wijayawardene N.N., Crous P.W., Kirk P.M., Hawksworth D.L., Boonmee S., Braun U., Dai D.-Q., D’souza M.J., Diederich P., Dissanayake A. (2014). Naming and outline of Dothideomycetes–2014 including proposals for the protection or suppression of generic names. Fungal Divers..

[22] Su H., Hyde K.D., Maharachchikumbura S.S.N., Ariyawansa H.A., Luo Z., Promputtha I., Tian Q., Lin C., Shang Q., Zhao Y. (2016). The families Distoseptisporaceae fam. nov., Kirschsteiniotheliaceae, Sporormiaceae and Torulaceae, with new species from freshwater in Yunnan Province, China. Fungal Divers..

[23] Liu J.W., Hu Y.F., Luo X.X., Castañeda-Ruíz R.F., Xia J.W., Xu Z.H., Cui R.Q., Shi X.G., Zhang L.H., Ma J. (2023). Molecular phylogeny and morphology reveal four novel species of *Corynespora* and *Kirschsteiniothelia* (Dothideomycetes, Ascomycota) from China: A checklist for *Corynespora* reported worldwide. J. Fungi.

[24] de Farias A.R.G., Afshari N., Silva V.S.H., Louangphan J., Karimi O., Boonmee S. (2024). Three novel species and new records of *Kirschsteiniothelia* (Kirschsteiniotheliales) from northern Thailand. MycoKeys.

[25] Hyde K.D., Jones E.B.G., Liu J.K., Ariyawansa H., Boehm E., Boonmee S., Braun U., Chomnunti P., Crous P.W., Dai D.Q. (2013). Families of Dothideomycetes. Fungal Divers..

[26] Nishi M., Okano I., Sawada T., Hara Y., Nakamura K., Inagaki K., Yaguchi T. (2018). Chronic *Kirschsteiniothelia* infection superimposed on a pre-existing non-infectious bursitis of the ankle: The first case report of human infection. BMC Infect. Dis..

[27] Guegan H., Cailleaux M., Le Gall F., Robert-Gangneux F., Gangneux J.P. (2021). Chromoblastomycosis due to a never-before-seen Dematiaceous fungus in a kidney transplant patient. Microorganisms.

[28] Matsushima T. (1996). Matsushima mycological memoirs 9. Matsushima Mycol. Mem..

[29] Liu J.K., Luo Z.L., Liu N.G., Cheewangkoon R., To-anun C. (2018). Two novel species of *Paradictyoarthrinium* from decaying wood. Phytotaxa.

[30] Htet Z.H., Prematunga C., Mapook A., Jones E.B.G., Kandawatte T.C. (2023). Taxonomy and phylogeny of *Paradictyoarthrinium salsipaludicola* sp. nov. (Paradictyoarthriniaceae, Pleosporales) from mangroves. Phytotaxa.

[31] Liu J.K., Hyde K.D., Jones E.B.G., Ariyawansa H.A., Bhat D.J., Boonmee S., Maharachchikumbura S.S.N., McKenzie E.H.C., Phookamsak R., Phukhamsakda C. (2015). Fungal diversity notes 1–110: Taxonomic and phylogenetic contributions to fungal species. Fungal Divers..

[32] Kirk P.M. (1982). New or interesting microfungi VI. *Sporidesmiella* gen.nov. (Hyphomycetes). Trans. Br. Mycol. Soc..

[33] Li X.H., Liu Y.L., Song H.Y., Hu D.M., Gao Y., Hu H.J., Zhou J.P. (2021). *Sporidesmiellalignicola* sp. nov., a new hyphomycetous fungus from freshwater habitats in China. Biodivers. Data J..

[34] Xiong Y.C., Xu R., Luo Z.L., Gao Q., Zhao Q. (2024). *Sporidesmiella motuoensis*, a new freshwater fungus from Tibetan Plateau, China. Phytotaxa.

[35] Crous P.W., Wingfield M.J., Schumacher R.K., Akulov A., Bulgakov T.S., Carnegie A.J., Jurjević Ž., Decock C., Denman S., Lombard L. (2020). New and interesting fungi. 3. Fungal Syst. Evol..

[36] Zhang H., Dong W., Hyde K.D., Maharachchikumbura S.S.N., Hongsanan S., Jayarama Bhat D., Al-Sadi A.M., Zhang D. (2017). Towards a natural classification of Annulatascaceae-like taxa: Introducing Atractosporales ord. nov. and six new families. Fungal Divers..

[37] Dong W. (2021). Towards a natural classification of Annulatascaceae-like taxa II: Introducing five new genera and eighteen new species from freshwater. Mycosphere.

[38] Jin W.T., Gernandt D.S., Wehenkel C., Xia X.M., Wei X.X., Wang X.Q. (2021). Phylogenomic and ecological analyses reveal the spatiotemporal evolution of global pines. Proc. Natl. Acad. Sci. USA.

[39] Su P.W., Lu Z.H., Tian W.H., Chen Y.P., Maharachchikumbura S.S.N. (2023). Six additions to the genus *Periconia* (Dothideomycetes: Periconiaceae) from graminaceous plants in China. J. Fungi.

[40] Tian W.H., Chen Y.P., Maharachchikumbura S.S.N. (2022). *Neodigitodesmium*, a novel genus of family Dictyosporiaceae from Sichuan Province, China. Phytotaxa.

[41] Tian W.H., Su P.W., Chen Y.P., Maharachchikumbura S.S.N. (2023). Four new species of *Torula* (Torulaceae, Pleosporales) from Sichuan, China. J. Fungi.

[42] Senanayake I.C., Rathnayaka A.R., Sandamali D.S., Calabon M.S., Gentekaki E., Lee H.B., Pem D., Dissanayake L.S., Wijesinghe S.N., Bundhun D. (2020). Morphological approaches in studying fungi: Collection, examination, isolation, sporulation and preservation. Mycosphere.

[43] Wanasinghe D.N., Phukhamsakda C., Hyde K.D., Jeewon R., Lee H.B., Gareth Jones E.B., Tibpromma S., Tennakoon D.S., Dissanayake A.J., Jayasiri S.C. (2018). Fungal diversity notes 709–839: Taxonomic and phylogenetic contributions to fungal taxa with an emphasis on fungi on Rosaceae. Fungal Divers..

[44] Toju H., Tanabe A.S., Yamamoto S., Sato H. (2012). High-Coverage ITS primers for the DNA-based identification of Ascomycetes and Basidiomycetes in environmental samples. PLoS ONE.

[45] White T.J., Bruns T.D., Lee S.B., Taylor J.W., Innis M.A., Gelfand D.H., Sninsky J. (1990). Amplification and Direct Sequencing of Fungal Ribosomal RNA Genes for Phylogenetics.

[46] Vilgalys R., Hester M. (1990). Rapid genetic identification and mapping of enzymatically amplified ribosomal DNA from several *Cryptococcus* species. J. Bacteriol..

[47] Cubeta M.A., Echandi E., Abernethy T., Vilgalys R. (1991). Characterization of anastomosis groups of binucleate *Rhizoctonia* species using restriction analysis of an amplified ribosomal RNA gene. Phytopathology.

[48] Hibbett D.S. (1996). Phylogenetic evidence for horizontal transmission of group I introns in the nuclear ribosomal DNA of mushroom-forming fungi. Mol. Biol. Evol..

[49] Rehner S.A., Buckley E. (2005). A Beauveria phylogeny inferred from nuclear ITS and *EF1*-alpha sequences: Evidence for cryptic diversification and links to Cordyceps teleomorphs. Mycologia.

[50] Jaklitsch W.M., Komon M., Kubicek C.P., Druzhinina I.S. (2005). *Hypocrea voglmayrii* sp. nov. from the Austrian Alps represents a new phylogenetic clade in *Hypocrea*/*Trichoderma*. Mycologia.

[51] Voglmayr H., Akulov O.Y., Jaklitsch W.M. (2016). Reassessment of *Allantonectria*, phylogenetic position of *Thyronectroidea*, and *Thyronectria caraganae* sp. nov. Mycol. Prog..

[52] Paradis E., Schliep K. (2019). ape 5.0: An environment for modern phylogenetics and evolutionary analyses in R. Bioinformatics.

[53] Katoh K., Misawa K., Kuma K., Miyata T. (2002). MAFFT: A novel method for rapid multiple sequence alignment based on fast Fourier transform. Nucleic Acids Res..

[54] Capella-Gutiérrez S., Silla-Martínez J.M., Gabaldón T. (2009). trimAl: A tool for automated alignment trimming in large-scale phylogenetic analyses. Bioinformatics.

[55] Lanfear R., Frandsen P.B., Wright A.M., Senfeld T., Calcott B. (2016). PartitionFinder 2: New methods for selecting partitioned models of evolution for molecular and morphological phylogenetic analyses. Mol. Biol. Evol..

[56] Nguyen L.T., Schmidt H.A., von Haeseler A., Minh B.Q. (2015). IQ-TREE: A fast and effective stochastic algorithm for estimating maximum-likelihood phylogenies. Mol. Biol. Evol..

[57] Huelsenbeck J.P., Ronquist F. (2001). MRBAYES: Bayesian inference of phylogenetic trees. Bioinformatics.

[58] Yu G. (2020). Using ggtree to Visualize Data on Tree-Like Structures. Curr. Protoc. Bioinform..

[59] Crous P.W., Groenewald J.Z. (2011). Why everlastings don’t last. Persoonia.

[60] Crous P.W., Schoch C.L., Hyde K.D., Wood A.R., Gueidan C., de Hoog G.S., Groenewald J.Z. (2009). Phylogenetic lineages in the Capnodiales. Stud. Mycol..

[61] Bao D.F., Luo Z.L., Liu J.K., Bhat D.J., Sarunyav N., Li W.L., Su H.Y., Hyde K.D. (2018). Lignicolous freshwater fungi in China III: Three new species and a new record of *Kirschsteiniothelia* from northwestern Yunnan Province. Mycosphere.

[62] Doilom M., Dissanayake A.J., Wanasinghe D.N., Boonmee S., Liu J.K., Bhat D.J., Taylor J.E., Bahkali A.H., McKenzie E.H.C., Hyde K.D. (2017). Microfungi on *Tectona grandis* (teak) in Northern Thailand. Fungal Divers..

[63] Crous P.W., Osieck E.R., Jurjević Ž., Boers J., van Iperen A.L., Starink-Willemse M., Dima B., Balashov S., Bulgakov T.S., Johnston P.R. (2021). Fungal Planet description sheets: 1284–1382. Persoonia.

[64] Pem D., Jeewon R., Chethana K.W.T., Hongsanan S., Doilom M., Suwannarach N., Hyde K.D. (2021). Species concepts of Dothideomycetes: Classification, phylogenetic inconsistencies and taxonomic standardization. Fungal Divers..

[65] Schoch C.L., Sung G.H., López-Giráldez F., Townsend J.P., Miadlikowska J., Hofstetter V., Robbertse B., Matheny P.B., Kauff F., Wang Z. (2009). The Ascomycota tree of life: A phylum-wide phylogeny clarifies the origin and evolution of fundamental reproductive and ecological traits. Syst. Biol..

[66] Wijayawardene N.N., Hyde K.D., Dai D.Q., Sanchez-Garcia M., Goto B.T., Saxena R.K., Erdoğdu M., Selcuk F., Rajeshkumar K.C., Aptroot A. (2022). Outline of Fungi and fungus-like taxa—2021. Mycosphere.

[67] Shenoy B.D., Jeewon R., Wu W.P., Bhat D.J., Hyde K.D. (2006). Ribosomal and *RPB2* DNA sequence analyses suggest that *Sporidesmium* and morphologically similar genera are polyphyletic. Mycol. Res..

[68] Mäkelä M.R., Donofrio N., de Vries R.P. (2014). Plant biomass degradation by fungi. Fungal Genet. Biol..

[69] Chen Y.P., Su P.W., Hyde K.D., Maharachchikumbura S.S.N. (2023). Phylogenomics and diversification of Sordariomycetes. Mycosphere.

[70] Maharachchikumbura S.S.N., Hyde K.D., Jones E.B.G., McKenzie E.H.C., Bhat D.J., Dayarathne M.C., Huang S.K., Norphanphoun C., Senanayake I.C., Perera R.H. (2016). Families of Sordariomycetes. Fungal Divers..

